# Characterization of zika virus infection of human fetal cardiac mesenchymal stromal cells

**DOI:** 10.1371/journal.pone.0239238

**Published:** 2020-09-17

**Authors:** Fiorella Rossi, Benjamin Josey, Ece Canan Sayitoglu, Renee Potens, Tolga Sultu, Adil Doganay Duru, Vladimir Beljanski

**Affiliations:** 1 NSU Cell Therapy Institute, Dr. Kiran C. Patel College of Allopathic Medicine, Nova Southeastern University, Fort Lauderdale, FL, United States of America; 2 Department of Molecular Biology and Genetics, Bogaziçi University, Istanbul, Turkey; 3 Science for Life Laboratory, Department of Medicine Solna, Karolinska Institutet, Stockholm, Sweden; University of Hong Kong, HONG KONG

## Abstract

Zika virus (ZIKV) is a single-stranded RNA virus belonging to the family Flaviviridae. ZIKV predominantly enters cells using the TAM-family protein tyrosine kinase receptor AXL, which is expressed on a range of cell types, including neural progenitor cells, keratinocytes, dendritic cells, and osteoblasts. ZIKV infections have been associated with fetal brain damage, which prompted the World Health Organization to declare a public health emergency in 2016. ZIKV infection has also been linked to birth defects in other organs. Several studies have reported congenital heart defects (CHD) in ZIKV infected infants and cardiovascular complications in adults infected with ZIKV. To develop a better understanding of potential causes for these pathologies at a cellular level, we characterized ZIKV infection of human fetal cardiac mesenchymal stromal cells (fcMSCs), a cell type that is known to contribute to both embryological development as well as adult cardiac physiology. Total RNA, supernatants, and/or cells were collected at various time points post-infection to evaluate ZIKV replication, cell death, and antiviral responses. We found that ZIKV productively infected fcMSCs with peak (~70%) viral mRNA detected at 48 h. Use of an antibody blocking the AXL receptor decreased ZIKV infection (by ~50%), indicating that the receptor is responsible to a large extent for viral entry into the cell. ZIKV also altered protein expression of several mesenchymal cell markers, which suggests that ZIKV could affect fcMSCs’ differentiation process. Gene expression analysis of fcMSCs exposed to ZIKV at 6, 12, and 24 h post-infection revealed up-regulation of genes/pathways associated with interferon-stimulated antiviral responses. Stimulation of TLR3 (using poly I:C) or TLR7 (using Imiquimod) prior to ZIKV infection suppressed viral replication in a dose-dependent manner. Overall, fcMSCs can be a target for ZIKV infection, potentially resulting in CHD during embryological development and/or cardiovascular issues in ZIKV infected adults.

## Introduction

Zika virus (ZIKV), a single-stranded RNA virus, belongs to the family Flaviviridae, genus Flavivirus [1]. ZIKV infection was first reported in Uganda in 1947; and since then, the virus has been sporadically found in Africa, Asia, and other continents [2]. Between 2013 and 2014, ZIKV was first identified in Brazil, and in 2015, it was reported spreading through the Americas and the Caribbean [3]. In 2016, The World Health Organization (WHO) declared the ZIKV epidemic a global health emergency due to the virus’s association with fetal microcephaly [4]. Evidence indicates that microcephaly and associated brain anomalies may be the most severe manifestations of the damage caused by ZIKV, but ZIKV infections may also result in a spectrum of developmental disorders known as Congenital Zika Syndrome (CZS). CZS is characterized by distinct features which include severe microcephaly (with partially collapsed skull), brain abnormalities, ocular abnormalities, congenital contractures, marked early hypertonia, symptoms of extrapyramidal involvement, and hearing loss [5,6]. While the full sequelae of damages caused by CZS has yet to be fully elucidated, most studies and clinical guidelines focus solely on the neurological impacts. Still, several studies have indicated that ZIKV infection may also pose a threat to the heart in both infants and adults. In early 2018, an infant was born with congenital heart defects to a mother with a confirmed case of ZIKV infection, and the child presented with hypoplastic left heart syndrome and other features of CZS including microcephaly [7]. Subsequently, three large studies reported increases in the occurrence of cardiac defects in infants with CZS [8–10]. For example, Noronha *et al* assessed heart pathology and reported minor cardiac pathology in two out of the five cases, but markers of ZIKV infection were not detected in the heart tissue [11]. Alternatively, Sousa *et al*. performed histologic evaluation of seven neonates with congenital ZIKV infections and did not find any abnormalities in the hearts, but did detect ZIKV in four out of the five infant heart tissues [12]. Despite the differences in these findings, the overall presence of cardiac complications was consistent in both studies. Moreover, several studies in adults have also presented strong evidence indicating an association between ZIKV infections and cardiac complications in the form of myocarditis, pericarditis, atrial fibrillation, cardiac arrhythmias, and heart failure [13–17]. Altogether, these observations suggest that heart tissue could be another target of ZIKV, however, potential cardiac-related effects of ZIKV infection have not been well-characterized.

One of the receptors most commonly implicated in ZIKV entry in neural progenitor cells is AXL receptor tyrosine kinase (AXL) [18]. AXL receptor (“anexelekto” in Greek means “uncontrolled”) is a member of the family composed of Tyro3, AXL, and Mer tyrosine (TAM) receptors [19,20]. ZIKV first binds to Growth arrest-specific 6 (Gas6) via phosphatidylserine on the viral membrane and then this complex binds to AXL [21]. This receptor is also expressed in many other cell types found in the nervous, cardiovascular, and immune systems, where it plays critical roles in normal development and function [19,22]. Currently, there is a limited understanding of the effects of ZIKV infection in other AXL expressing cells of stem/stromal cell origin. Beys-da-Silva *et al*. investigated the proteomics of ZIKV infection in adult adipose mesenchymal stromal cells (MSCs) [1], which are multipotent adult stromal cells with a capacity to differentiate into osteocytes, osteoblasts, chondrocytes, and adipocytes [23,24]. Mumtaz, *et al*. showed that ZIKV infection perturbs osteoblast-derived MSC function without causing cytopathic effects, and additionally found a delay in osteoblast development and maturation compared to uninfected controls [25]. To determine whether ZIKV can infect and replicate in cells relevant to cardiac pathophysiology, we utilized fetal cardiac mesenchymal stromal cells (fcMSCs), which were previously isolated from fetal hearts of six to nine-week-old embryos [26]. Cardiac MSCs represent a major population of non-myocyte heart cells. Embryologically, they originate from either the pro-epicardium or migratory neural crest, and depending on their heritage, can contribute to the morphological determination, mechanical integrity, and electrical conduction in the cardiac structure [27]. The fcMSCs used in this study were previously characterized as multipotent cardiac progenitor cells, which can give rise to endothelial cells, cardiomyocytes, and vascular smooth muscle cells [28,29].

In this study, we demonstrated that not only does ZIKV productively infect fcMSCs, predominantly via the AXL receptor, but also causes cell death. Additionally, Zika infection of fcMSCs triggers up-regulation of IFN-mediated antiviral responses. We also demonstrated that the upregulation of antiviral responses using TLR agonists poly-IC or imiquimod prior to ZIKV infection decreased ZIKV replication in fcMSCs. Overall, our results provide novel insight into mechanisms(s) potentially contributing to the development of ZIKV-related congenital heart defects and adult cardiac complications.

## Material and methods

### Cell culture

fcMSCs were obtained from 6–9 week old fetal hearts after the donor's informed consent, which was approved by the Regional Ethics Board in Stockholm (ethics approval number 2015/1369-31/2) as described previously [26]. Briefly, cells were cultured in 0.1% gelatin (Sigma) coated flasks/plates in DMEM/F12 media supplemented with 2% FBS (HyClone), 2mM L-glutamine (Gibco), 1x B27 cell culture supplement (Gibco), and MycoZap (Lonza) in the presence of 1 μl/ml of Wnt3a and 1 μl/ml of EGF (R&D SYSTEMS). Cells were maintained at 37°C in 5% CO_2_ and split every other day until plated at the appropriate cell concentration depending on the experimental setup. Detailed procedures and authentication data can be found in **S1 Fig**.

### Virus production and infection

ZIKA virus Puerto Rican strain (PR-VABC59) was purchased from ATCC (cat: VR-1843). Virus was produced by infecting Vero cells (obtained from ATCC, catalog number: CCL-81). ZIKV titers were determined by plaque assay and established as 1.7x10^7^ plaque forming units/ml (PFU/ml). For ZIKV infection, fcMSCs from four different donors (unless stated otherwise) were plated at a density of 3x10^5^ cells/well in 6-well plates and infected at different Multiplicity of Infection (MOI) of 0.1, 1, or 10 for 6 h unless specified otherwise. After the incubation, viral inoculum was removed, and cells were washed with 1X PBS. Finally, fresh media was replenished for a total of 24, 48, 72, and 96 h post-infection.

### Cell viability assays

Cell viability was assessed using CellTiter-Glo Luminescent Cell Viability Assay (Promega, cat: G7570). This assay determines the number of viable cells in culture based on quantitation of the ATP present, an indicator of metabolically active cells. Briefly, fcMSCs were seeded onto 0.1% gelatin coated 96-well plates at 5,000 cells/well in 100 μL of media and maintained at 37°C in 5% CO_2_ overnight prior to the initiation of treatments. For small molecule and antibody inhibitor assays, cell culture media was aspirated and replaced with fresh media containing inhibitors or DMSO (dimethyl sulfoxide), and cells were pre-treated for 1 h prior to infection. For viral toxicity evaluation, cells were infected with 1 MOI of virus at 37°C in 5% CO_2_ for 6 h, after which the media was removed, cells washed gently with Hank’s Balanced Salt Solution (HBSS), and the media replaced with standard culture media (above). At the indicated time points post-infection, an equal volume of CellTiter-Glo® 2.0 Reagent equal to the volume of cell culture medium present in each well was added, the contents were mixed for two minutes on an orbital shaker to induce cell lysis, and plates were incubated at room temperature for 10 minutes to stabilize the luminescent signal. Luminescence signal was record using FilterMax F5 plate reader (Molecular Devices).

### qRT-PCR

Primers were designed using the NCBI (National Center for Biotechnology) Primer Blast, (http://www.ncbi.nlm.nih.gov/tools/primer-blast/index.cgi?LINK_LOC=BlastHome). Exon-spanning primers capable of detecting multiple transcript variants were selected when possible. Detailed primer information can be found in **S2 Table**. Primers were synthesized by Midlands Certified Reagents (Midland, TX). After infection of fcMSCs, total RNA was isolated with E.Z.N.A. HP Total RNA kit by OmegaBio-tek (cat: R6812-02). After quality control and quantification via Nanodrop, cDNA was generated with qScript cDNA SuperMix by Quantabio (cat: 95048–025) per manufacturer’s instructions. cDNA mixture was quantified on Nanodrop, and all samples were normalized to 500 ng input. qRT-PCR was performed on the Agilent Aria MX using PerfeCTa SYBR Green FastMix (Quantabio, cat: 95072–250).

### Flow cytometry

All antibodies for flow cytometry experiments were purchased form BD Biosciences unless stated otherwise. Data were acquired on a BD LSR Fortessa X-20 flow cytometer and analyzed using FlowJo software v.10. Gating was performed based on first size (FSC vs SSC), then single cells, and after that live cells were gated for detailed phenotyping or for showing infection. Dilutions and conjugations for all antibodies are outlined in **S1 Table**. To detect live cells, non-infected or ZIKV-infected samples were stained first with live/dead aqua (ThermoFisher, cat: L34957) following manufacturer’s instructions. For surface AXL expression analysis, fcMSCs were stained with either AXL-APC antibodies (R&D Systems; clone 108724; cat: MAB154) or mouse IgG1 isotype control antibodies (R&D Systems; cat: MAB002). For the phenotyping panel, surface staining was done with antibodies for CD31 (clone WM59, cat: 550389), CD45 (clone HI30, cat: 555483), CD73 (clone AD2, cat: 550257), CD90 (clone 5E10, cat: 555595), CD106 (clone 51-10C9, cat: 555645), CD140a (clone αR1, cat: 556002), and CD172a (clone SE5A5, cat: 565036). After surface staining, cells were fixed and permeabilized with the fixation and permeabilization buffers of the BD Human Foxp3 Buffer Set (cat: 560098) following manufacturer’s instructions. Intracellular staining for ZIKV was performed using AF647-conjugated Flavivirus group antigen Antibody [D1-4G2-4-15 (4G2)] (Novus Biologicals, cat: NBP2-52666). This antibody binds to the fusion loop at the extremity of domain II of protein E of ZIKV. Moreover, cells were also stained for intracellular expression of ISL-1 (clone Q11-465, cat: 562547) and cardiac Troponin-T (clone 13–11, cat: 564767).

### Immunofluorescence

To determine the intracellular ZIKV replication, fcMSCs were seeded onto 0.1% gelatin coated 96-well clear bottom, black wall plates (Corning, cat#3904) at 5,000 cells/well in 100 μL of medium and maintained at 37°C in 5% CO_2_ overnight prior to the initiation of treatments. fcMSCs (n = 3) were infected with 1 MOI ZIKV for 48 h; cells were subsequently fixed with 4% PFA, permeabilized with 70% ethanol and stained using anti-Flavivirus group antigen antibody (D1-4G2-4-15) conjugated with Alexa Fluor 647 at 2 μg/ml (Novus Biologicals). After incubation, cells were stained with DAPI (Life technologies, USA) per manufacturer’s instructions. The intensity of ZIKV-positive signal in fcMSCs is proportional to viral replication and was assessed by automated fluorescence microscopy on a Thermo CX7 (ThermoFisher) [25].

### AXL neutralization assay

To evaluate the effect of AXL receptor blocking on ZIKA virus infection, fcMSCs from four different donors were plated at a density of 300,000 cells/well in 0.1% gelatin coated 6 well plates and cultured overnight. The following day, cells were pre-treated with 10 μg/ml of goat anti-human AXL polyclonal antibody (R&D SYSTEMS, AF154) or goat IgG isotype control (R&D SYSTEMS, AB-108-C) for 1 h prior to ZIKA virus infection (1 MOI for 6 h). This antibody has been previously reported to inhibit AXL-mediated ZIKV entry into other cell types at the indicated concentration [30]. AXL receptor blockade was maintained during the 6 h infection. After virus exposure, media was aspirated, cells washed with 1X PBS, and fresh media containing anti-AXL antibody or isotype control was added until analysis. Finally, cells were collected 48 h post-infection for RNA extraction and qRT- PCR was performed for the quantification of ZIKA virus replication.

### RNA sequencing

fcMSCs were seeded in 0.1% gelatin-coated six-well plates at a density of 1 × 10^5^ cells/well and infected with ZIKV at MOI 1 for 6, 12, and 24 h; after which the media was aspirated from cells, and cells were detached from wells using RNAzol reagent (Molecular Research Center) and stored at −80°C until RNA isolation. RNA was purified using RNAzol according to the manufacturer’s instructions. Total RNA (50 ng) was submitted to the Nova Southeastern University (NSU) Genomic Core, and 150 base paired-end read sequencing was performed using Illumina Next-Seq 500. All procedures were performed according to the manufacturer’s instructions. Quality control assessment was done using the Illumina RNAseq pipeline to estimate genomic coverage, percent alignment, and nucleotide quality. Raw sequencing data were transformed to fastq format and the remaining analysis was performed using CLC Genomics Workbench v9. Raw data were imported using paired reads with minimum distance of one and maximal distance of 1,000 nucleotides while failed reads were removed. Mapping to gene regions only was conducted using genome annotated reference sequence hg38 (Homo sapiens) with auto-detection of paired distances and the maximum number of hits for a read of 10. For both infected and uninfected samples at 6 and 12 h and for uninfected samples at 24 h approximately 96% to 98% of all reads were mapped successfully. However, we were not able to map 29% to 48% of reads for ZIKV-infected samples at 24 h likely due to the presence of high levels of viral nucleic acids.

### Toll-like receptor activation

fcMSCs progenitor cells were plated at a density of 3 x 10^5^ cells/well in 0.1% gelatin coated 6 well-plates. Cells were pre-treated for 1 h with 5 μg/ml of poly I:C (Sigma-Aldrich) or 5 μg/ml of imiquimod (Enzo Life Sciences), then cells were infected with 0.1, 1, or 10 MOI of ZIKV. ZIKV replication was assessed using qRT-PCR for ZIKV NS1 mRNA 48 h post infection. For TLR dose-dependent activation, fcMSCs were pretreated with 0.1, 1, or 10 μg/ml of either poly I:C or imiquimod, cells were then infected with 0.1 MOI of ZIKV for 6 h. Cells were harvested 72 h post-infection, fixed, permeabilized, and intracellular staining of ZIKV was quantified by flow cytometry as described above. Following virus exposure period in both experiments, media was aspirated, cells were washed with 1X PBS, and fresh media added.

#### Statistical analysis

Statistical analyses were performed using GraphPad Prism (GraphPad Sofware Inc., La Jolla, CA, Version 8). Data are expressed as means +/- standard errors of the mean (S.E.M) unless otherwise indicated. One-way analysis of variance (One way-ANOVA) test was used to determine statistical significance between each treatment group, if present. *p* values ≤0.05 were considered statistically significant.

## Results

### ZIKV infects and replicates in fcMSCs

**Fig 1:**
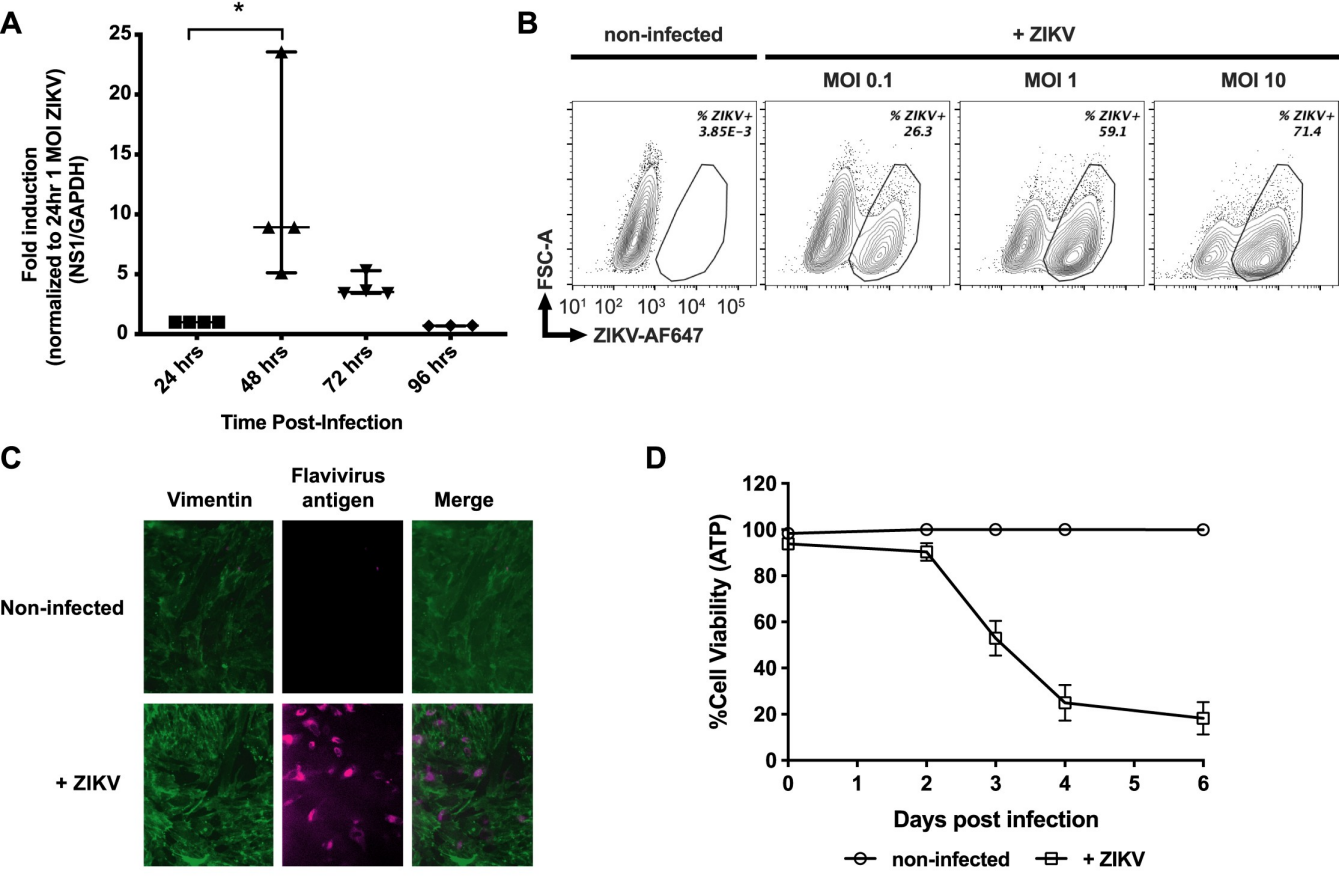
ZIKA virus infects and kills fetal cardiac progenitor cells. fcMSCs (n = 4) were infected with 1 MOI of ZIKV for 6 h. (A) fcMSCs were assessed for NS1 mRNA levels 24, 48, 72 and 96 h post-infection using qRT-PCR. Median values are plotted with 95% CI. (B) Intracellular staining for ZIKV detection was performed with anti-flavivirus group antigen 4G2 antibody 48 h post-infection, as described in Materials and Methods. Representative image shown. For flow cytometry data acquisition, aqua live/dead stained cells were first gated for their size (FSC vs SSC) and then the percentage of ZIKV infected live cells was determined using 4G2 antibody. (C) Immunofluorescence staining for group antigen 4G2 was performed at 48 h post-infection using confocal microscopy. (D) ZIKV-infected fcMSCs (n = 4 donors) and their matched non-infected controls were pre-treated as indicated and cell viability was assessed using the CellTiter-Glo Luminescent Cell Viability Assay, which determines cell viability based on quantification of intracellular ATP.

To evaluate whether ZIKV can productively infect fcMSCs, we exposed fcMSCs from n = 4 donors to ZIKV (MOI of 1) and determined viral replication by evaluating non-structural protein 1 (NS1) mRNA levels by qRT-PCR at various time points (24, 48, 72, and 96 h). The peak of ZIKV replication occurred at 48 h, reaching about 17-fold higher compared to 24 h post-infection (**Fig 1A**). At 96 h post-infection, we detected approximately 15-fold reduction compared to the peak of infection at 48 h in levels of NS1 mRNA, possibly resulting from cell death. In agreement with qRT-PCR data, flow cytometry analysis of ZIKV infection at different MOIs at 48 h showed a direct correlation between viral load and percentage of fcMSCs infected (**Fig 1B**). This was confirmed by immunofluorescence detection of Flavivirus group antigen in ZIKV infected fcMSCs 48 h post-infection (**Fig 1C**).

To evaluate the relationship between cell viability and ZIKV infection, we infected fcMSCs with ZIKV and followed cell viability for six days. We observed that fcMSCs began dying 48 h post-infection, as indicated by a decrease in cell viability (**Fig 1D**), and at four days (96 h) post-infection, about 80% of the cells were non-viable (**Fig 1D**). These results indicate that ZIKV reaches a peak of replication at 48 h post-infection, resulting in a progressive decrease in fcMSC viability. This leads to a decrease in intracellular ZIKV levels at later time points resulting in inverse correlation between ZIKV infection and cell death in fcMSCs.

### ZIKV alters expression of mesenchymal origin markers

**Fig 2:**
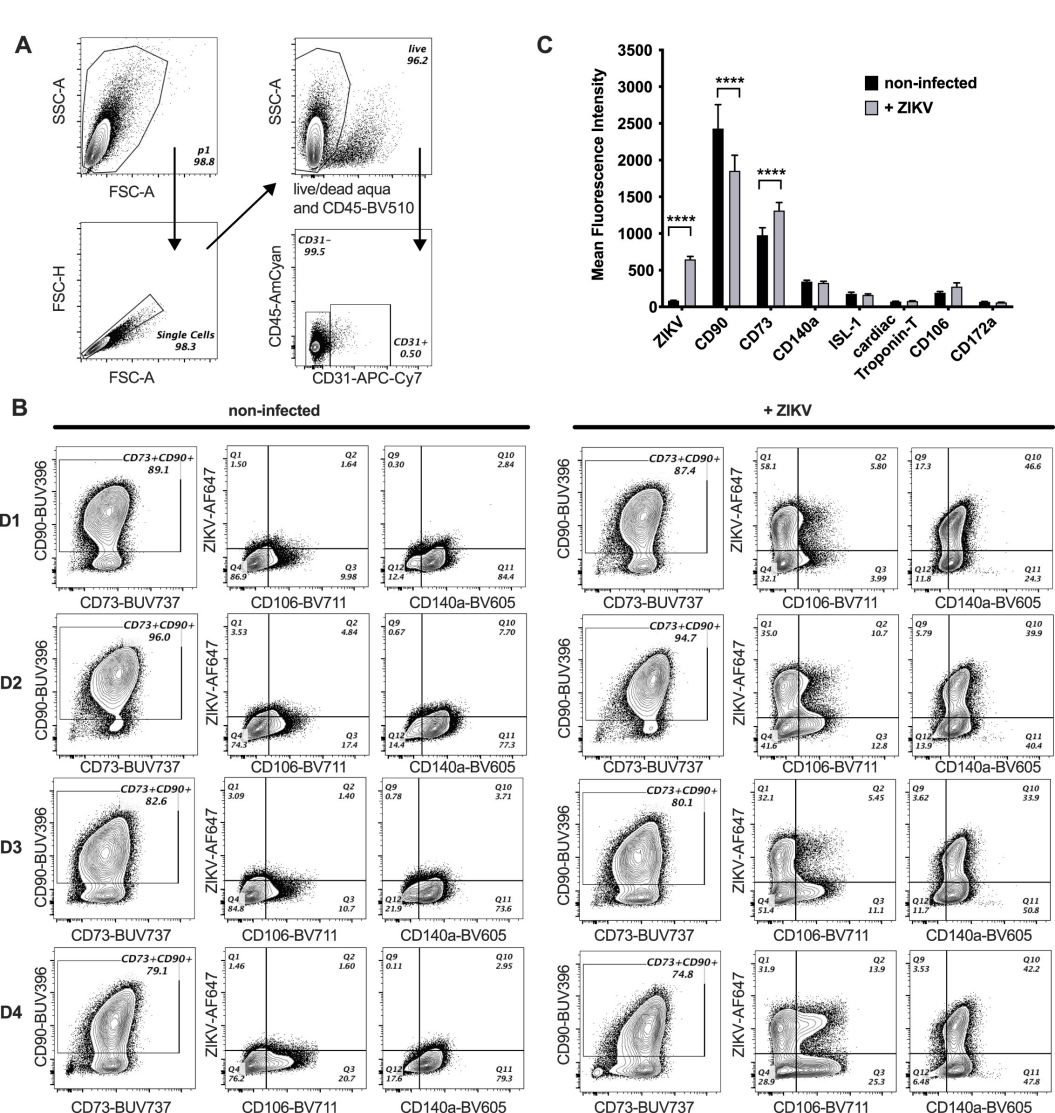
ZIKV infection alters expression of mesenchymal markers. fcMSCs (n = 4) were infected or not with 1 MOI ZIKV for 6 h. Cell surface staining was performed 48 h post-infection using antibodies against CD31, CD45, CD73, CD90, CD106, CD140a and CD172a; and intracellular staining with 4G2 antibody (for ZIKV detection), ISL-1, and cardiac Troponin-T. (A) fcMSCs were selected for their size (FSC vs SSC), viability, and subsequently CD45^−^CD31^−^ cells were used to assess expression of selected markers. (B) Flow cytometry assessment of indicated markers expression, with and without ZIKV infection. Mean MFI of all markers for all donors tested are plotted with error bars indicating SEM. D1—D4: donors 1–4. (C) Bar graphs depicting mean fluorescence intensity (MFI) for different markers in ZIKV infected cells (grey bars) or non-infected controls (black bars) with ZIKV as indicated.

fcMSCs were previously characterized for increased expression of cardiac progenitor markers, ISL1, OCT4, KDR, NKX2.5, and PDGFR-α (CD140a), whereas sub-populations also expressed the progenitor markers TBX18, KDR, c-KIT, and SSEA-1 [26]. fcMSCs can arise from two distinct lineages in the embryo: the migratory neural crest and the pro-epicardium. We observed mRNA profiles of lineage tracing markers in these fcMSCs to be consistent with those of pro-epicardial origin (**S1 Fig**). To determine whether ZIKV infection affects expression of cardiac-associated and mesenchymal origin cell surface markers, fcMSCs were infected with 1 MOI ZIKV for 6 h, and the expression of CD90, CD73, CD140a (PDGFRa), ISL-1, CD172a (SIRPa), cardiac Troponin-T (cardiomyocytes), CD106 (VCAM-1, vascular smooth muscle cells), and CD31 (PECAM-1, endothelial cells) were evaluated by flow cytometry 48 h post-infection (**Fig 2**). We observed very low percentages of cells expressing either the hematopoietic marker CD45 (<3%) or the terminal endothelial cell marker CD31 (<1%). These cells were excluded from MSC analysis. We found that several mesenchymal, but not cardiac progenitor, markers changed their expression after infection. For example, the expression of CD90 decreased about 23% in ZIKV-infected compared to un-infected cells. A reduction of this marker has been associated with an increased differentiation capacity of MSCs [31]. Conversely, the expression of CD73 was up-regulated by approximately 34% compared to non-infected cells (**Fig 2C**). The other MSC and cardiac markers, CD140a, ISL-1, cardiac Troponin-T, CD106, and CD172a did not change expression levels upon infection with ZIKV. Altogether, this indicates that ZIKV infection can alter fcMSC phenotype, which could impact fcMSC differentiation capacity.

### ZIKA virus enters fcMSCs via both AXL and additional mechanism(s)

**Fig 3:**
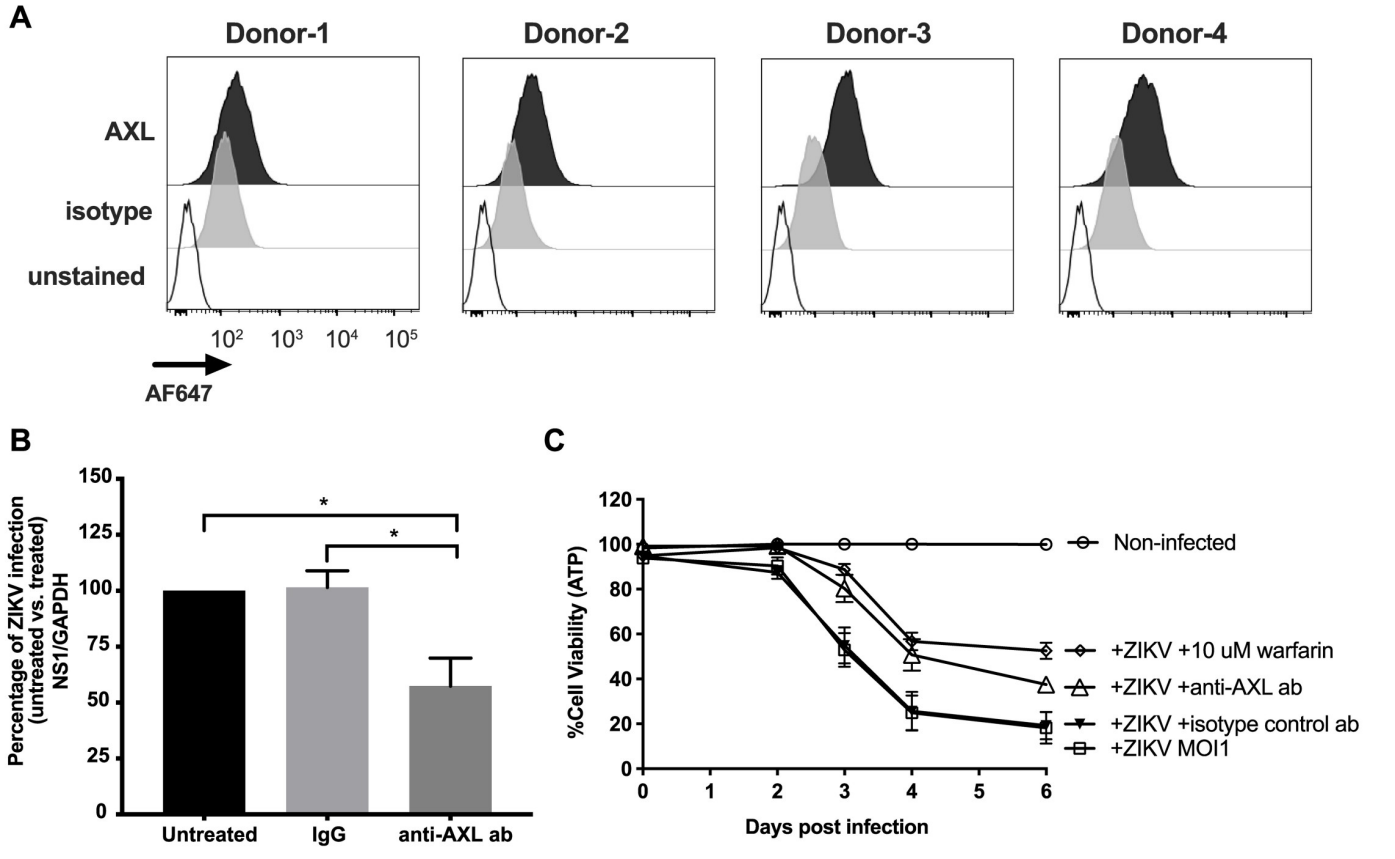
AXL antibody decreases ZIKV infection. (A) fcMSCs (donors 1–4) were stained with either isotype control or anti-AXL-APC antibodies as indicated on the panel and AXL expression was evaluated using flow cytometry. (B) fcMSCs were pre-treated with AXL blocking antibody or isotype control for 1 h and infected with MOI 1 of ZIKV for 6 h. Assessment of ZIKV replication in fcMSCs was performed by qRT-PCR quantification of NS1 mRNA levels 48 h post-infection. (C) fcMSCs were pre-treated with anti-AXL antibody (10 μg/mL), isotype control antibody, or warfarin and infected for 1 h prior to infection with 1 MOI ZIKV for 6 h, maintaining pre-treatment throughout duration, and cell viability was assessed at the indicated time points post-infection using the CellTiter-Glo Luminescent Cell Viability Assay.

To investigate whether AXL receptor plays a role in ZIKV’s entry into fcMSCs, we first measured surface expression of AXL on fcMSCs by flow cytometry. Similar to observations in other ZIKV target cells, we found that fcMSCs express AXL receptor (**Fig 3A**). To assess whether AXL receptor mediates ZIKV entry and infection, we treated fcMSCs (n = 4) with AXL blocking antibody for 1 h prior to infection with ZIKV 1 MOI for 6 h. As a negative control, we treated matched cells with isotype control antibody at the same concentration and treatment conditions. Total RNA was collected from fcMSCs 48 h post-infection to assess ZIKV replication by quantifying ZIKV NS1 mRNA using qRT-PCR. We found that pre-treatment of fcMSCs with AXL-blocking antibody decreased levels of NS1 mRNA levels approximately 50% compared to untreated and isotype controls (**Fig 3B**), which suggests that AXL receptor is involved in ZIKV entry into fcMSCs. Since we observed only partial suppression of ZIKV infection with AXL blockade, other TAM receptors may also mediate ZIKV entry into fcMSCs [32]. To further validate our findings, we assessed cell viability using intracellular ATP measurements in fcMSCs that were treated with anti-AXL antibody for 1 h prior to ZIKV infection. In addition, we also pre-treated matched cells with 10 μM of the pan-TAM receptor antagonist warfarin [33] in parallel with anti-AXL antibody. Because pre-treatment of fcMSCs with either anti-AXL antibody or warfarin only partially decreased cytotoxic effect of the virus, additional mechanisms involved in ZIKV entry/infection in these cells must also exist.

### ZIKV infection-driven changes in gene expression

**Fig 4:**
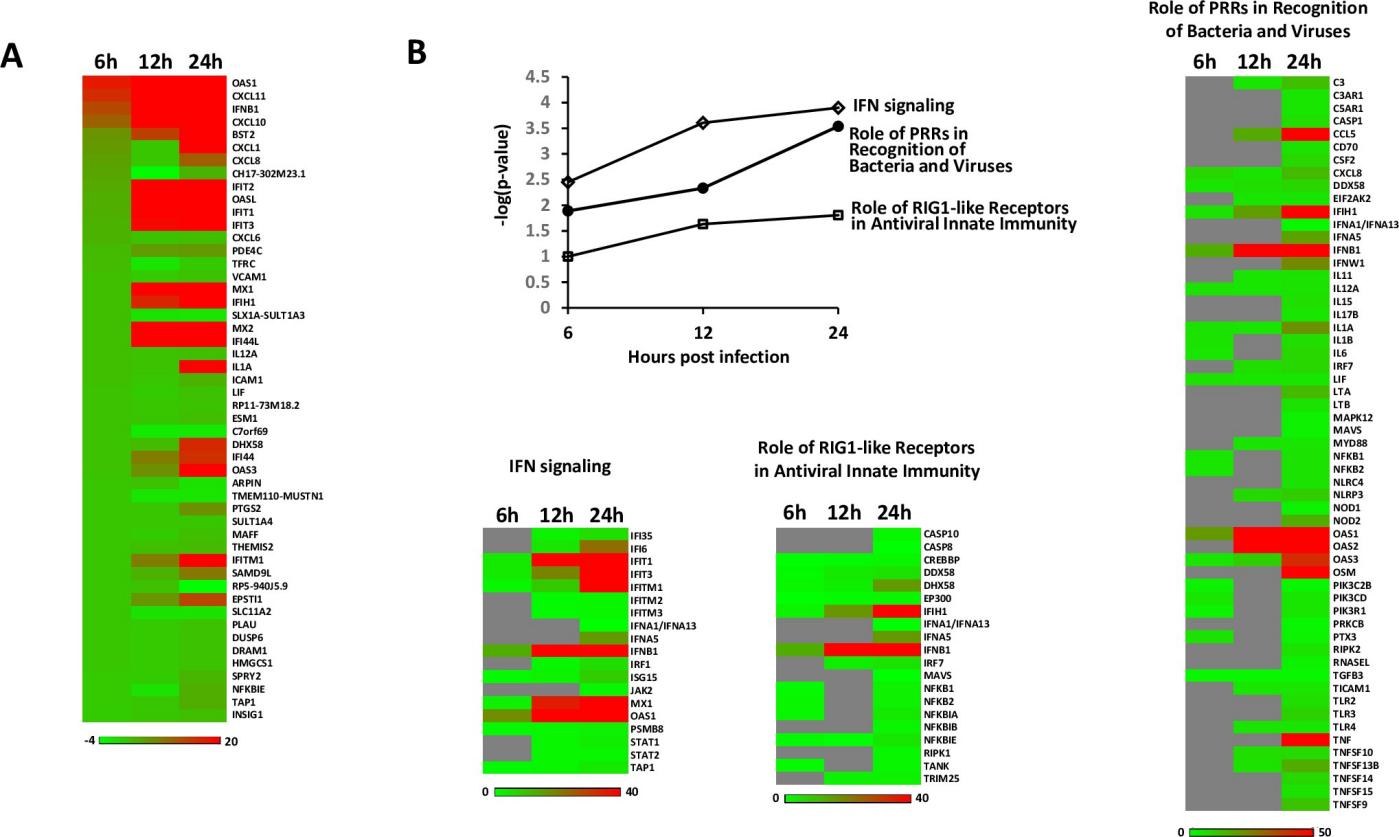
Gene expression analysis of fcMSCs infected with ZIKV for 6, 12, or 24 h. fcMSCs (n = 4) were infected with ZIKV for indicated times and RNA sequencing was performed as described in Materials and Methods. (A) Heatmap representation of the expression for top abundant transcripts; (B) Selected pathway activation at 6, 12, and 24 h post-infection is expressed as–log(-p-value) which takes into account both levels and numbers of differentially expressed genes that are assigned to the pathway. The differentially expressed genes belonging to the three selected antiviral pathways are represented in heatmaps. The expression intensities in all heatmaps is represented using green-red color scale with the scale bar at the bottom. Gray color indicates differentially regulated genes that did not reach statistical significance.

Our findings revealed that the peak of ZIKV replication in fcMSCs occurs 48 h post-infection and also initiates cell death. To gain insight into the early cellular responses prior to the 48 h replication peak, fcMSCs were infected with ZIKV MOI 1 for 6, 12, and 24 h. Immediately following infection, total RNA was isolated, and RNA sequencing (RNA-Seq) was performed. Differentially regulated genes (DRGs, fold changes > 2.0, false discovery rate < 0.05%) were uploaded into Ingenuity Pathway Analysis (IPA) software, and the top differentially regulated genes and pathways underwent functional clustering using IPA software. The majority of genes upregulated due to ZIKV infection increased between 6 and 24 h. The majority of the top 50 DRGs are those known to be induced by IFN (**Fig 4A**). Canonical pathway analysis using Ingenuity Pathway Analysis software identified pro-inflammatory pathways such as IFN signaling, role of PPRs in recognition of bacteria and viruses, and role of RIG1-like receptors in antiviral innate immunity as the main up-regulated functional categories (**Fig 4B**). Subsequent kinetic analysis revealed that ZIKV infection of fcMSCs induces increase in IFN signaling between 6–24 h. Such results are in line with the first and second wave of antiviral response where antiviral factors produce antiviral factors, including sets of IFN-regulated genes. This continuous upregulation of IFN signaling subsequently triggers synthesis of genes and pathways which provide additional defense mechanisms against viral replication [34].

### Stimulation of TLR pathways decrease ZIKV infection/replication

**Fig 5:**
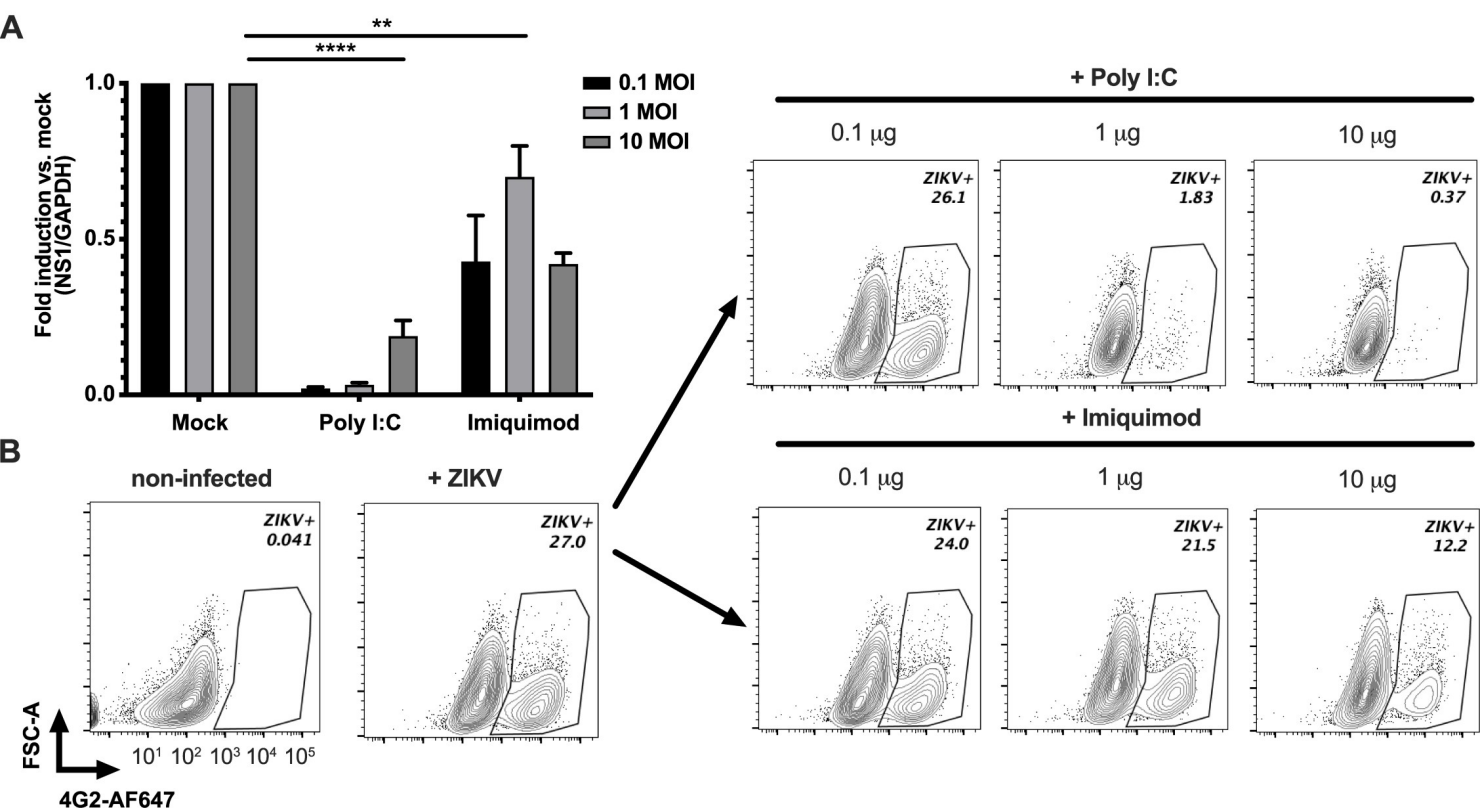
TLR agonists decrease ZIKV replication. (A) fcMSCs from one donor were pre-treated for 1 h with 5 μg/ml of either poly I:C or imiquimod and subsequently infected with 0.1, 1, or 10 MOI of ZIKV. Viral replication was assessed using qRT-PCR for NS1 mRNA 48 h post infection. (B) fcMSCs were pre-treated with multiple concentrations of TLR3 and TLR7 agonists (0.1, 1, or 10 μM), and infected with 0.1 MOI of ZIKV for 6 h. ZIKV replication was assessed via flow cytometry by staining with 4G2 antibody 72 h post-infection.

Previous findings indicate that stimulation of toll-like receptors (TLRs) such as TLR3, TLR7, TLR8, and TLR9 induce a potent response against viruses known to induce type I IFN response(s) [35–40]. Based on our gene expression findings, ZIKV-induced antiviral response in fcMSCs peaks at 24 h post-infection leading to upregulation of the highest number of IFN-stimulated genes and pathways. To gain preliminary insight into whether stimulation of IFN response prior to ZIKV infection can decrease or block viral replication, we pre-treated fcMSCs from one of the donors for 1 h with TLR3 or TLR7 agonists poly I:C or imiquimod, respectively. Subsequently, fcMSCs were infected with ZIKV at 0.1, 1, or 10 MOIs for 6 h. ZIKV replication was assessed by qRT-PCR to assess NS1 protein mRNA levels 48 h post-infection. Our findings indicate that pre-treatment of the cells with poly I:C almost completely abrogated infection at 0.1 and 1 MOI, and significantly reduced at 10 MOI ZIKV (**Fig 5A**). While pre-treatment of cells with imiquimod partially reduced ZIKV replication at 0.1 MOI, only a slight reduction in viral replication was detected at MOIs higher than 0.1, suggesting that imiquimod pre-treatment is only efficient in blocking ZIKV replication at lower MOI. (**Fig 5A**). In addition, we observed a dose-dependent decrease of ZIKV in cells pre-treated with the agonists (**Fig 5B**). Although these findings are preliminary, they suggest that activation of the TLR3 pathway might be used to off-set some of the effects associated with ZIKV infection.

## Discussion

In this study, we aimed to determine the ability of ZIKV to infect and replicate in fcMSCs and to gain insight into the response of fcMSCs to ZIKV infection. While the full range of their biological functions is still not completely understood, many studies have shown fcMSCs to play important role(s) in both normal and pathophysiological cardiac development and function. We hypothesized that ZIKV infection-mediated elimination of these cells or modulation of their multipotency has the potential to trigger cardiac abnormalities (short-term and long-term). We report that ZIKV infected and replicated in fcMSCs with a peak of infection at 48 h followed by cell death at 72 h. Our findings suggest that ZIKV utilizes the TAM receptor AXL to enter fcMSCs, in addition to other entry mechanisms. Differential gene expression analysis in infected cells revealed upregulation of type I IFN response to ZIKV, with a peak at 12 h.

As mentioned previously, several groups reported both the presence of ZIKV in post-mortem cardiac tissues, as well as cardiovascular dysfunction in both ZIKV infected infants and adults. These cells were previously characterized as a heterogeneous population of cells demonstrating multipotent cardiogenic potential with limited capability to form cardiomyocytes [41]. In this study, we further determined that the cells expressed mRNA profiles consistent with fcMSCs derived primarily from the pro-epicardium. While the involvement of epicardial progenitors in CHD and adult cardiomyopathies is not clearly defined, many studies have indicated roles for this region in both cardiac development and response to injury, including the hypoplastic left heart syndrome observed in the ZIKV infant case study [42,43]. fcMSCs have been characterized for both their mesenchymal and cardiac lineages. Our findings suggest that ZIKV infection and replication has the potential to induce cardiac issues through induction of cell death and modulation of phenotype in cardiac MSCs, as well as induction of a pro-inflammatory response in cardiac tissue.

To evaluate whether ZIKV infection impacts fcMSC differentiation potential, we examined both cardiac and mesenchymal origin marker expression after ZIKV infection. While we did not observe changes in expression of cardiac markers, the expression of MSC markers CD90 and CD73 were observed to alter in infected cells. We found that ZIKV infection decreased expression of CD90, and such decrease was previously associated with an increase in differentiation capacity for MSCs [31]. This suggests that ZIKV-induced increase in CD90 could “push” fcMSCs toward differentiation. On the other hand, we found an increase in CD73 receptor, an ecto-5-prime-nucleotidase enzyme which, together with CD39, converts extracellular ATP to adenosine. CD73 has previously been associated with reductions in local immune responses and immune escape mechanisms [44–47]. This ZIKV-mediated increase in CD73 could facilitate viral replication by allowing the virus to evade host immune response in cardiac tissue.

Numerous studies have consistently shown that AXL receptor facilitates ZIKV entry in neural progenitor cells. fcMSCs also express AXL receptor on their surface, and our results indicate involvement of AXL in ZIKV entry into these cells. Specifically, ZIKV replication in fcMSCs decreased to approximately 50% compared to control when AXL was blocked with an antibody. These results align with several studies indicating that TAM receptors are the main entry receptors for ZIKV.

ZIKV was reported to induce inflammatory responses with an increased secretion of pro-inflammatory cytokines IL-1β, IL-6, MIP1α, and chemokines such as IP-10 and RANTES [48], and an effective antiviral response against ZIKV is mediated via activation of type I IFN pathway. In line with these findings, our gene expression analysis revealed that a number of genes with roles in innate immune response were upregulated along with ZIKV infection/replication. We observed changes in mRNA levels of genes known to be involved in the innate antiviral response, a subset of which was found to be upregulated at 6 h, with peak upregulation occurring at 12 h to 24 h post-infection. We also found that IFN signaling pathway and cytosolic pattern recognition pathways also peaked at 12–24 h. This is consistent with the kinetics of IFN antiviral responses frequently seen in other viral infections. Consistent with our gene expression data, stimulation of TLR response with TLR3 and TLR7 agonists, showed almost complete suppression of infection for TLR3 stimulation and partial suppression of infection for TLR7 stimulation in one of the fcMSC donors. Additional *in vivo* data will be required in order to fully evaluate the potential of TLR agonists to suppress ZIKV replication and decrease ZIKV-induced pathologies.

In summary, our findings indicate that ZIKV efficiently infects and replicates in fcMSCs. This ability of ZIKV to infect cardiac MSCs could be related to cardiac issues observed in infants born to ZIKV infected mothers, as well as in adults infected with ZIKV. Additionally, our findings demonstrate that induction of type I IFN response in cardiac MSCS could be utilized prophylactically to decrease or even block some of the ZIKV-induced cellular damage.

## Supporting information

S1 Fig Characterization of fcMSCs for mRNA expression of MSC and cardiac progenitor markers. fcMSCs were characterized according to their markers of origin as indicated on the panel. Markers of MSC and cardiac origin were detected by qRT-PCR to validate the use of these cells.
(PPTX)

S1 Table Antibodies and dilutions used for flow cytometry analysis. (PDF)

S2 Table List of primer sequences used to phenotype fcMSCs. (PDF)

## Nonstandard abbreviations and acronyms

CHD: congenital heart defects
CZS: Congenital Zika Syndrome
Gas6: Growth arrest-specific 6
PFU: plaque forming unit
HBSS: Hank’s Balanced Salt Solution
FSC: forward scatter
SSC: side scatter
MFI: mean fluorescence intensity
MOI: multiplicity of infection
NS1: non-structural protein 1
RNA-seq: RNA sequencing
DRG: differentially regulated genes
TLR: Toll-like receptor
ZIKV: Zika Virus
fcMSCs: fetal cardiac mesenchymal stem cells
MSCs: mesenchymal stromal cells
TAM: (TYRO, AXL and MERK)
