# Correction: Characterization of zika virus infection of human fetal cardiac mesenchymal stromal cells

**DOI:** 10.1371/journal.pone.0246112

**Published:** 2021-01-22

**Authors:** Fiorella Rossi, Benjamin Josey, Ece Canan Sayitoglu, Renee Potens, Tolga Sutlu, Adil Doganay Duru, Vladimir Beljanski

The fifth author's name is spelled incorrectly. The correct name is: Tolga Sutlu. The correct citation is: Rossi F, Josey B, Sayitoglu EC, Potens R, Sutlu T, Duru AD, et al. (2020) Characterization of zika virus infection of human fetal cardiac mesenchymal stromal cells. PLoS ONE 15(9): e0239238. https://doi.org/10.1371/journal.pone.0239238

## References

[1] Rossi F, Josey B, Sayitoglu EC, Potens R, Sultu T, Duru AD, et al (2020) Characterization of zika virus infection of human fetal cardiac mesenchymal stromal cells. PLoS ONE 15(9): e0239238 10.1371/journal.pone.0239238 32941515 PMC7498051

