# Supplementary figures and images for: Characterization of zika virus infection of human fetal cardiac mesenchymal stromal cells
Source: PLoS One. 2020 Sep 17;15(9):e0239238. doi: 10.1371/journal.pone.0239238 (PMC7498051; doi:10.1371/journal.pone.0239238)

## Slide 1
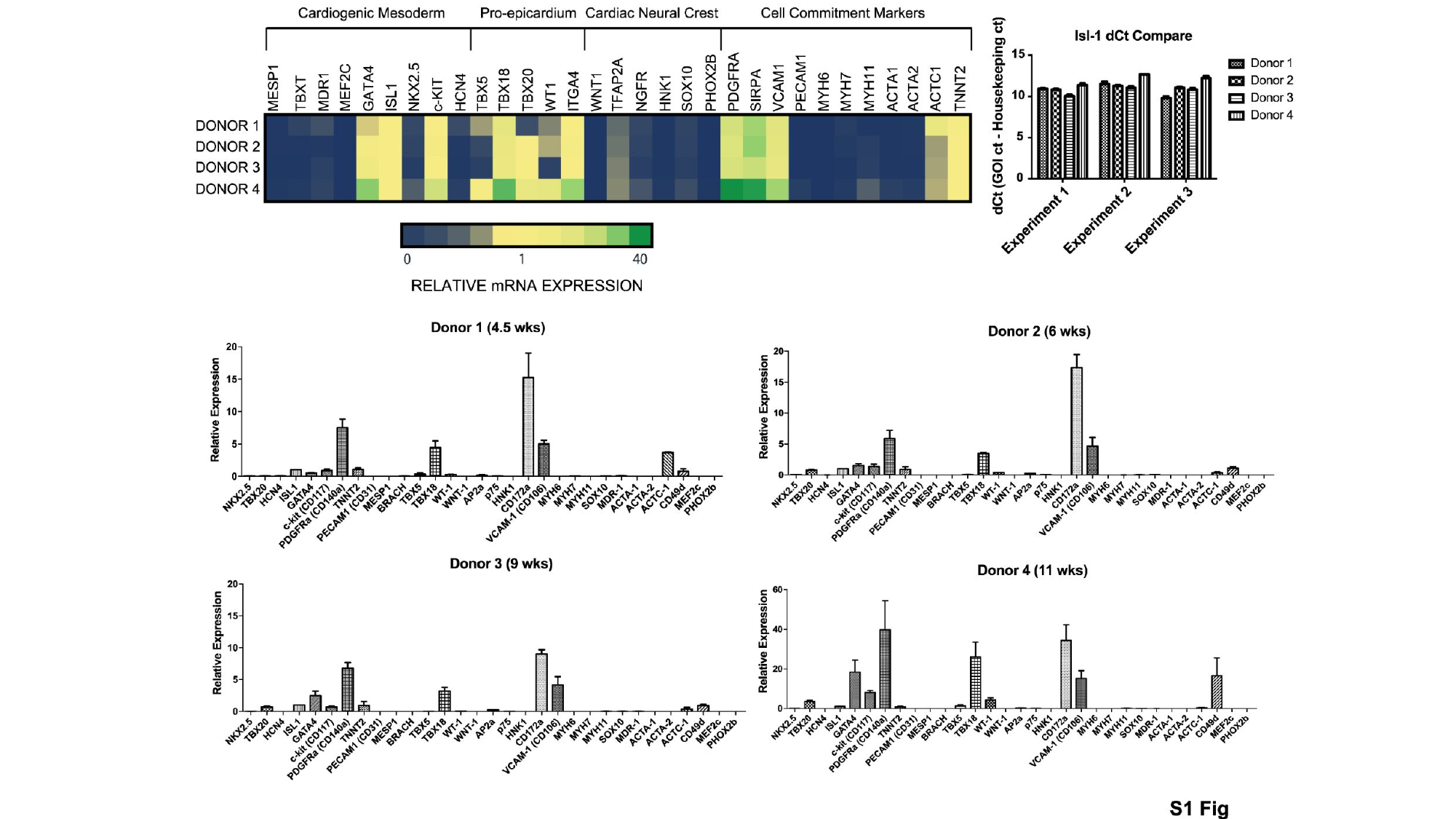

Supplement: S1 Fig — fcMSCs were characterized according to their markers of origin as indicated on the panel. Markers of MSC and cardiac origin were detected by qRT-PCR to validate the use of these cells. (PPTX) [file pone.0239238.s001.pptx]
