# Supplementary material for: Characterization of zika virus infection of human fetal cardiac mesenchymal stromal cells
Source: PLoS One. 2020 Sep 17;15(9):e0239238. doi: 10.1371/journal.pone.0239238 (PMC7498051; doi:10.1371/journal.pone.0239238)
Supplement: S1 Table — (PDF) [file pone.0239238.s002.pdf]

Supplementary Table 1. Antibodies and dilutions used for flow cytometry analysis

Antibody	Supplier	Volume used per test*
AXL-APC	BD Biosciences	5ul
CD31-APC-Cy7	BD Biosciences	2.5ul
CD45-AmCyan	BD Biosciences	2.5ul
CD73-BUV737	BD Biosciences	2.5ul
CD90-BUV395	BD Biosciences	2.5ul
CD106-BV711	BD Biosciences	2.5ul
CD140a-BV605	BD Biosciences	2.5ul
CD172a-FITC	BD Biosciences	2.5ul
ZIKV flavigroup 4G2-AF647	Novus Biologicals	7.5ul
ISL-1-PE	BD Biosciences	5ul
Cardiac Troponin-T-PE	BD Biosciences	5ul

* test means $1x10^6$ cells, up to 200 ml volume
