# Supplementary material for: Characterization of zika virus infection of human fetal cardiac mesenchymal stromal cells
Source: PLoS One. 2020 Sep 17;15(9):e0239238. doi: 10.1371/journal.pone.0239238 (PMC7498051; doi:10.1371/journal.pone.0239238)
Supplement: S2 Table — (PDF) [file pone.0239238.s003.pdf]

Supplementary Table 2. List of primer sequences used to phenotype fcMSCs

Target	Accession # (s) *=multiple transcript variants detected	Primer Sequence	Amplicon Size
Zika NS1		*Fwd* - TGGAGTTCAACTGACGGTCG<br>*Rev* - TACCCCGAACCCATGATCCT	241
AXL	NM_021913.4*	*Fwd* - GTTTGGAGCTGTGATGGAAGGC<br>*Rev* - CGCTTCACTCAGGAAATCCTCC	121
GAPDH	NM_002046.6*	*Fwd* - AAGGTCGGAGTCAACGGATTT<br>*Rev* - TGAAGGGGTCATTGATGGCA	100
MESP1	NM_018670.3	*Fwd* - CGAGTCCTGGATGCTCTCTG<br>*Rev* - CCATGAGTCTGGGGACGAGA	100
TBX5	NM_000192.3	*Fwd* - CCACAAACCAACCTTGAGCTG<br>*Rev* - GTTCTGCTCTCCAACTATCCCA	178
TBX20	NM_001077653.2*	*Fwd* - AGGAGGCGACGGAGAACA<br>*Rev* - CTGCCCGACTTGGTGATG	286
HCN4	NM_005477.2	*Fwd* - GACTGCTGGGTGTCCATCAA<br>*Rev - AGAGCGCGTAGGAGTACTGCTT*	67
ISL1	NM_002202.2	*Fwd* - TACAAAGTTACCAGCCACC<br>*Rev* - GGAAGTTGAGAGGACATTGA	157
BRACHYURY (TBXT)	NM_003181.3	*Fwd* - ACGATCCTGGGTGTGCGTA<br>*Rev* - GACCAAGACTGTCCCCGCTC	221
GATA4	NM_001308093.1*	*Fwd* - CGGAAGCCCAAGAACCTGA<br>*Rev* - CTGCTGTGCCCGTAGTGAG	176
MEF-2c	NM_002397.4*	*Fwd* - GGGTATGGCAATCCCCGAAA<br>*Rev* - GGTCGACATCCTCAGACACTG	184
c-KIT (CD117)	NM_000222.2*	*Fwd* - TGGGCCACCGTTTGGAAAGCT<br>*Rev* - AGGGTGTGGGGATGGATTTGCTCT	168, 156
PDGFRα (CD140a)	NM_006206.5*	*Fwd* - ATCAATCAGCCCAGATGGAC<br>*Rev* - TTCACGGGCAGAAAGGTACT	891
α-SMA (ACTA2)	NM_001141945.2	*Fwd* - AAAGCAAGTCCTCCAGCGTT<br>*Rev* - CAGGATTCCCGTCTTAGTCCC	194
FLK-1/KDR (CD309)	NM_002253.3	*Fwd* - TCTGGCTACTTCTTGTCATCATCC<br>*Rev* - TGGCATCATAAGGCAGTCGTTC	141
PECAM1 (CD31)	NM_000442.4	*Fwd* - CAGGCTCCCACTGGCCTGACT<br>*Rev* - TGCCCTTGCGGTGTTAGGCA	184
α-skeletal actin (ACTA1)	NM_001100.3	*Fwd* - CCCGCCCAGAAACTAGACAC<br>*Rev* - GTCGCCCACGTAGGAATCTT	195
α-cardiac actin (ACTC1)	NM_005159.4	*Fwd* - CCGTACCACAGGCATTGTTC<br>*Rev* - GACAAAGGAGTAGCCACGCT	166
TNNT2	NM_001001431.2*	*Fwd* - AGAAGGCCAAGGAGCTGTGGCA<br>*Rev* - CCAGCGCCCGGTGACTTTAGC	170
NKX2.5	NM_004387.3	*Fwd* - CACCGGCCAAGTGTGCGTCT<br>*Rev* - CACCGGCCAAGTGTGCGTCT	117
MDR1	NM_001348945.1	*Fwd - AAATGGCTACATGAGAGCGG*<br>*Rev - TAGTGGAAAGACCACAGATGACT*	231
WT1	NM_000378.5	*Fwd - CGCTATTCGCAATCAGGGTTAC*<br>*Rev - ATGAGTGGTTGGGGAACTGC*	101

Supplementary Table 2. List of primer sequences used to phenotype fcMSCs

Target	Accession # (s) *=multiple transcript variants detected	Primer Sequence	Amplicon Size
WNT1	NM_005430.3	*Fwd - CCTCCACGAACCTGCTTACA*	247
		Rev - GTTTCTCGACAGCCTCGGTT	
SOX10	NM_006941.3	*Fwd - TCTGGAGGCTGCTGAACGAAAG*	202
		Rev - TTGTAGTGGGCCTGGATGGC	
SIRPA	NM_001040022.1*	*Fwd - AAATACCGCCGCTGAGAACA*	211
		Rev - TGTGATATCATTTGTGTCCTGTGT	
MYH6	NM_002471.3	*Fwd - TCTCCGTGAAGGGATAACCA*	246
		Rev - ACAGTCACCGTCTTCCCATTC	
MYH11	NM_002474.2	*Fwd - CCGGGAAAACCGAAAACACC*	103
		Rev - TTTTCCAGCTCTCCCGTGAT	
MYH7	NM_000257.3	*Fwd - ACACACTTGAGTAGCCCAGG*	242
		Rev - TTCACGGTCACTGTCTTGCC	
TFAP2A	NM_003220.2	*Fwd - GAGTAGCTCCACTTGGGTGC*	115
		Rev - GTGACGGTCCTCGCAGTC	
NGFR	NM_002507.3	*Fwd - TGGATTACACGGTCCACACC*	248
		Rev - CTGTTCCACCTCTTGAAGGCT	
HNK1	NM_018644.3	*Fwd - GGGTTGTGAGTGCTGGTAAT*	125
		Rev - GTGCCAGACAGTGATGAGCA	
SOX10	NM_006941.3	*Fwd - TCTGGAGGCTGCTGAACGAAAG*	202
		Rev - TTGTAGTGGGCCTGGATGGC	
PHOX2B	NM_003924.3	*Fwd - TACGCCGCAGTTCCTTACAAA*	121
		Rev - CGAAGACCCTTTCCAGCTCTT	
VCAM1	NM_001078.4*	*Fwd - TTGGATAATGTTTGCAGCTTCTCA*	190
		Rev - AGATGTGGTCCCCTCATTCGT	
ACTA2	NM_001141945.2	*Fwd - AAAGCAAGTCCTCCAGCGTT*	194
		Rev - CAGGATTCCCGTCTTAGTCCC	
